# Henoch-Schönlein Purpura with Adalimumab Therapy for Ulcerative Colitis: A Case Report and Review of the Literature

**DOI:** 10.1155/2016/2812980

**Published:** 2016-07-27

**Authors:** Joseph J. LaConti, Jean A. Donet, Jeong Hee Cho-Vega, Daniel A. Sussman, Dana Ascherman, Amar R. Deshpande

**Affiliations:** ^1^Division of Rheumatology, University of Miami Leonard Miller School of Medicine, Miami, FL 33136, USA; ^2^Division of Gastroenterology, University of Miami Leonard Miller School of Medicine, Miami, FL 33136, USA; ^3^Department of Pathology, University of Miami Leonard Miller School of Medicine, Miami, FL 33136, USA

## Abstract

Tumor necrosis factor-*α* (TNF*α*) inhibitor therapy has signified an important milestone in the fight against many rheumatological disorders and inflammatory bowel disease (IBD). Cutaneous adverse events caused by this class of medications are well known but relatively uncommon. Most reactions are mild and rarely warrant treatment withdrawal. Henoch-Schönlein purpura (HSP) is a disease with cutaneous vasculitis, arthritis, and gastrointestinal and renal involvement that is usually seen in children, though the worst complications are typically seen in adults. We present a case of HSP complicating adalimumab treatment in a patient with ulcerative colitis who had achieved endoscopic remission. We review similar cases reported in the literature and discuss the consequences of these autoimmune diseases.

## 1. Introduction

Tumor necrosis factor alpha (TNF*α*) inhibitors have widespread use in patients with rheumatological and gastrointestinal conditions, especially inflammatory bowel disease, where they have been shown to improve symptoms, lead to bowel mucosal healing, reduce hospitalizations and surgeries, and spare corticosteroid use [1]. A wide variety of adverse events including infections, malignancies, and cutaneous reactions have been reported with use of these medications. Cutaneous side effects are found in 29% of patients with IBD while receiving anti-TNF*α* treatment, but discontinuation of therapy is rarely required [2]. Small vessel vasculitis is a rare contributor to the cutaneous adverse effects seen during anti-TNF*α* treatment, and the majority of cases show resolution with drug discontinuation [3]. Henoch-Schönlein purpura (HSP) is an acute vasculitic syndrome presenting with cutaneous purpura, arthritis, and gastrointestinal and renal impairment generally seen in children; it has rarely been associated with anti-TNF*α* treatment. Here we present a case of an adult male with ulcerative colitis who developed HSP while being treated with adalimumab.

## 2. Case Presentation

**Figure 1:**
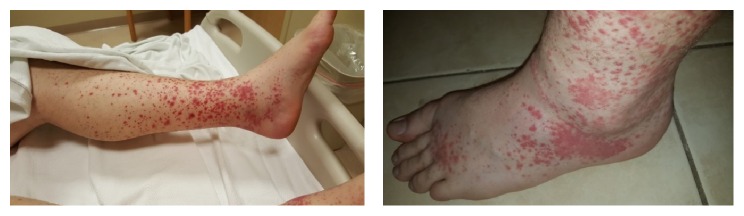
Palpable purpura on the lower extremities with ankle edema and arthritis.

A 33-year-old Caucasian man with ulcerative colitis for three years in endoscopic and near histologic remission three months before, on adalimumab 40 mg every two weeks (Abbvie, North Chicago, IL), with therapeutic trough levels and no antidrug antibodies, was admitted to our hospital for workup of a recurrent erythematous, palpable, nonblanching rash on his bilateral lower extremities associated with joint pain and swelling of his ankles, knees, and elbows (Figure 1). He first noticed this rash two months before and was treated with empiric oral antibiotics, with incomplete resolution. The rash then recurred with more severity and ascended to his buttocks, lower back, and abdomen; biopsy at an outside facility was suggestive of a superficial perivascular dermatitis. His adalimumab was stopped and he was treated with a week of oral steroids which resulted in resolution of the rash. However, when his oral steroids were completed, his rash reappeared in the same locations, and he was subsequently admitted to our hospital for lack of response of the rash thus far. He denied any fevers, chills, night sweats, weight loss, abdominal pain, change in bowel habits, gross hematuria, or blood in the stools since the onset of the rash. He denied recent respiratory, genitourinary, or gastrointestinal infections, recent travel, sick contacts, or exposure to new foods or medications. In his family history, he has two uncles with chronic kidney disease but the patient did not know the etiology. He had no allergies. The patient was a married male with no smoking or alcohol use history.

**Figure 2:**
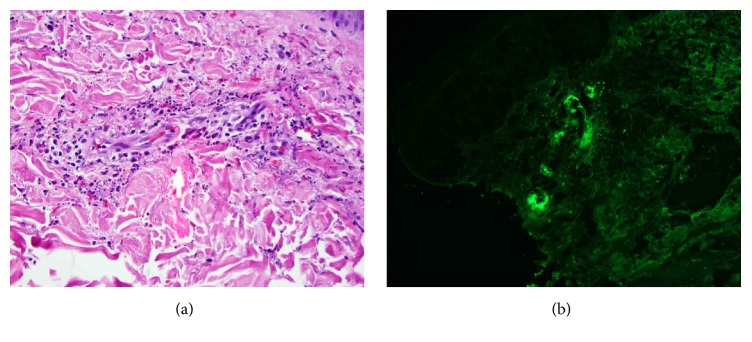
(a) High power hematoxylin and eosin stained slide showing leukocytoclastic vasculitis. (b) Direct immunofluorescence showing superficial dermal vascular depositions of IgA.

On physical exam, a palpable purpuric rash was present on his bilateral lower extremities from his toes up to his knees and then less prominently on his upper thighs along with several scattered lesions on his abdomen up to his umbilicus, buttocks, and lower back. Ankles were mildly tender and swollen without other signs of synovitis. Abdominal examination was otherwise normal. Laboratory data was significant for a mild leukocytosis of 11,600 (90% neutrophils), mild acute kidney injury with creatinine of 1.11 mg/dL, and slight proteinuria and hematuria; hemoglobin, platelet count, liver chemistries, and ESR were normal. HIV, viral hepatitis serologies, antinuclear antibody, antineutrophil cytoplasmic antibody (ANCA), cryoglobulins, and complement levels were all negative or within normal limits. Punch skin biopsies from the patient's right lower extremity revealed leukocytoclastic vasculitis with frequent eosinophils and direct immunofluorescence was positive for small vessel IgA deposition (Figure 2).

On the basis of these findings, the patient was diagnosed with HSP. Without a clear infectious trigger, we concluded that this episode was likely related to anti-TNF*α* treatment with adalimumab. The patient received treatment with methylprednisolone 20 mg intravenously every eight hours with almost complete resolution of the rash. His renal injury resolved as well. He was discharged on an oral prednisone taper. Several weeks later he had persistent resolution of the rash off steroids and adalimumab. The patient has been seen in follow-up every three months by either the gastroenterology or rheumatology team. The initial treatment of steroids and cessation of adalimumab resulted in resolution of his purpura and arthritis. He had no proteinuria on urinalysis. He continued to have up to 10 nonbloody bowel movements per day. A repeat colonoscopy showed mild, focal, active colitis. He is being treated with mesalamine with good control of his symptoms.

## 3. Discussion

We describe here an unusual case of HSP in an adult patient who had been treated with adalimumab for ulcerative colitis. Without any other clear trigger for the development of HSP, we attribute this to the use of anti-TNF*α* therapy.

The annual incidence of HSP ranges in different reports from 13 to 20/100,000 for children and infants. The incidence is much less for adults, closer to 1-2/100,000. The disease is often described as seasonal due to the fact that cases increase during the fall and winter months, when a preceding infection of the respiratory tract is more likely to act as the trigger. The classic findings comprise a tetrad of nonthrombocytopenic palpable purpura, arthritis, abdominal pain, and renal involvement such as proteinuria or hematuria. The arthritis is usually symmetric and has a predilection for the ankles and knees. Involvement of the gastrointestinal tract can range from symptoms of cramping and pain to the more feared complications of intussusception, hemorrhage, or perforation, which are more commonly seen in infants and children. Skin involvement is common and is a nearly universal sign of the disease. Crops of nonblanching purpura develop predominately in the dependent areas of the lower extremities and buttocks. The classic finding on skin biopsy is leukocytoclastic vasculitis with IgA deposits in the vasculature on direct immunofluorescence, similar to what was seen on the skin biopsy of our patient [4].

In adult patients with HSP, involvement of the kidney must be identified early, as an increased proportion of adults have persistent hematuria, proteinuria, or renal failure relative to children. While our patient initially had microhematuria and modest increase in creatinine, both of these parameters were corrected with administration of intravenous fluids and corticosteroids.

Our patient did not have clear evidence of a preceding infection to trigger the development of HSP. Group A beta-hemolytic* Streptococcus* (GAS) was seen in 20–50% of patients with acute HSP. Other pathogens such as parvovirus B19,* Bartonella henselae*,* Staphylococcus aureus*,* Helicobacter pylori*,* Haemophilus parainfluenzae*, Coxsackievirus, adenovirus, hepatitis A, and hepatitis B have also been implicated as infectious triggers of the disease [4]. However, the evidence linking these pathogens to the development of HSP consists of small reports of a descriptive nature and a true pathophysiological link has yet to be established.

Our patient was being treated with the TNF*α* blocker adalimumab for his ulcerative colitis. There are rare reports that have highlighted a case similar to ours, where a patient without a clear inciting event developed HSP while receiving an antagonist of TNF*α*. A 19-year-old male with Crohn's disease being treated with adalimumab developed purpura, joint pain, proteinuria, and hematuria. A skin biopsy showed leukocytoclastic vasculitis, consistent with HSP. Adalimumab was stopped as a precaution and then restarted with the rationale that this agent had no previous evidence to link it to HSP development. Upon reinitiation of the agent, the patient's symptoms returned [5]. In a similar case, a 36-year-old woman with Crohn's disease on adalimumab developed purpura, arthritis, proteinuria, and hematuria, with evidence of leukocytoclastic vasculitis on biopsy. Adalimumab was stopped and her symptoms resolved, but on rechallenge with the agent in order to better control her Crohn's disease the multisystem effects recurred [6].

This association with HSP is not unique to adalimumab, extending to other members of the TNF*α* inhibitor class. In two separate reports, a twelve-year-old female and a 69-year-old female both developed HSP while being treated with infliximab for ulcerative colitis [7, 8]. Beyond these cases, a sixty-year-old female with rheumatoid arthritis developed purpura and hematuria while receiving etanercept; both skin and kidney biopsies showed IgA and C3 deposition diagnostic of HSP. This patient also saw resolution of her disease with cessation of etanercept, only to have the signs and symptoms reappear when etanercept was restarted [9]. Overall, this limited but seemingly growing body of literature suggests that many members of the TNF*α* inhibitor class may illicit an HSP reaction. Interestingly, however, there have been reports of patients with TNF*α* inhibitor-induced HSP being treated with a different TNF*α* without recurrence of HSP [6].

More generally, it has been long known that skin reactions can be seen during treatment with TNF*α* antagonists. In an analysis of a Spanish database of 5437 patients treated with etanercept, infliximab, or adalimumab representing 17,300 years of exposure, the most common reactions were psoriasiform changes, alopecia areata, cutaneous lupus, vitiligo, lichenoid eruption, morphea, granuloma annulare, and vasculitis [10]. Similarly, in a review of published reports of autoimmune diseases that developed while taking TNF*α* targeted therapy, 118 cases of vasculitis were found among 379 diagnoses of a new autoimmune disease. Of these 118 cases, 102 of the cases had cutaneous involvement, 44 were documented as leukocytoclastic vasculitis, and two were confirmed HSP cases [11]. Other reports include a 37-year-old female who developed leukocytoclastic vasculitis while being treated with golimumab for ankylosing spondylitis [12], a 20-year-old male who developed leukocytoclastic vasculitis while being treated with adalimumab for Crohn's and juvenile idiopathic arthritis [13], and a 30-year-old female who developed cutaneous vasculitis while being treated with infliximab for juvenile idiopathic arthritis [14]. Cumulatively these data underscore the fact that skin reactions are not limited to a specific TNF*α* inhibitor, gender, or diagnosis that required TNF*α* inhibition.

While autoimmune reactions such as HSP are rare among patients being treated with TNF*α* antagonists, these cases raise the question whether there is a pathophysiological link between the inhibition of TNF*α* and the cascade that leads to deposition of IgA complexes in skin and other organs. While genetic factors may predispose certain individuals to the development of HSP, large scale genome-wide association studies have not been performed in patients with this disease, and the evidence that has been reported thus far has not provided a link between specific genetic changes and risk of HSP. The current literature offers some interesting candidates that compromise pathways already linked to other autoimmune and inflammatory conditions (CTLA-4, HLA gene family, TLR-2, TLR-4, IL-1, IL-6, and IL-18) [15].

Beyond these genetic considerations, the observation that many patients who developed HSP while receiving TNF*α* antagonists were being treated for inflammatory bowel disease is provocative. IBD and HSP involve a breach of similar mucosal tissues (gut and respiratory mucosa, resp.), though IgA has not typically been considered a major player in the predominately T cell mediated pathogenesis of IBD. However, recent work suggested that differential coating of bacteria by IgA can help identify those organisms most likely to cause colitis [16]. Studies such as this and the clinical reports featured above suggest that more scientific inquiry into the role of IgA in autoimmune diseases such as HSP and IBD is warranted.
